# University Students as Change Agents for Health and Sustainability: A Pilot Study on the Effects of a Teaching Kitchen-Based Planetary Health Diet Curriculum

**DOI:** 10.3390/nu16040521

**Published:** 2024-02-13

**Authors:** Nicola Rosenau, Uwe Neumann, Stacey Hamblett, Thomas Ellrott

**Affiliations:** 1 Institute for Nutrition and Psychology at the Georg-August-University Göttingen, University Medical Centre, Humboldtallee 32, 37073 Göttingen, Germany; 2 Culinary Medicine Germany e.V., 48341 Altenberge, Germany; uwe.neumann@culinarymedicine.de; 3 The Teaching Kitchen Collaborative, 101 Middlesex Turnpike, Suite 6, Burlington, MA 01803, USA; staceyhamblett@teachingkitchens.org

**Keywords:** education for sustainable development, sustainable development goals, plant-based diets, sustainable diets, planetary health literacy, teaching kitchens, cooking skills, cooking competencies, food-based dietary guidelines, reformulation of recipes

## Abstract

Global dietary habits are one of the main drivers of climate change. At the same time, they contribute to 11 million premature deaths every year. This raises the question of how the urgently needed transformation of food systems can be realized. Regardless of their degree paths, all university students, in their role as potential future experts and leaders in their fields, can serve as important change agents in society. In this paper, we (a) introduce a university curriculum in a teaching kitchen setting that is based on the planetary health diet (PHD) of the EAT-Lancet Commission, (b) investigate its feasibility, and (c) analyze its effects on the planetary health diet literacy of a pilot cohort of university students enrolled in various degree programs. We developed seven flipped classroom teaching kitchen sessions based on social cognitive theory (SCT), each consisting of a one-hour seminar with student presentations on various nutrition- and sustainability-related key topics, followed by corresponding two-hour hands-on cooking classes. To assess feasibility, specific questions from the official teaching evaluation of the University of Göttingen were analyzed. Changes in self-assessed planetary health diet literacy were measured using a pre- and post-survey. During the pilot phase, 26 students successfully completed the course. A total of 25 participants responded to the teaching evaluation and expressed high satisfaction with the course, the learning outcomes, and the level of demand. A total of 26 participants completed the pre- and post-survey. At the post-intervention, the students rated their planetary health diet literacy as 21 to 98% higher than before their course participation. The findings of this pilot study indicate that the curriculum was well-received and feasible with the target group, and they demonstrate that the course participation increased the university students’ self-assessed ability to disseminate strategies for more sustainable and healthy diets. Through replication at other universities worldwide, the teaching kitchen-based planetary health diet curriculum might foster a social shift towards healthier and more climate-friendly food systems.

## 1. Introduction

Current global eating habits are not sustainable and are harmful to both the planet and human health. Western diets in particular, with a high proportion of animal-based foods, make a major contribution to greenhouse gas emissions [1,2] and are responsible for significant changes in land use, usage of freshwater resources [2,3], and over-application of nitrogen and phosphorus into natural systems [2]. At the same time, a high consumption of red meat and low consumption of vegetables and fruit are associated with many health risks, which leads to an increasing prevalence of diet-related diseases [4].

With the introduction of the planetary health diet (PHD) in 2019, the EAT-Lancet Commission has made a concrete suggestion of a global dietary concept that preserves both natural systems and human health. Developed by international experts, the PHD provides specific quantity recommendations for individual food groups, the adherence to which is intended to enable the nutrition of 10 billion people by 2050 while staying within the planetary boundaries. The PHD is mainly plant-based and emphasizes only a low proportion of animal-origin foods [5]. 

There is a growing body of literature that proves the positive impacts of dietary changes to more plant-based diets, as suggested by the EAT-Lancet Commission [6,7,8,9]. For example, a Swedish study concluded that a high accordance of the individual diet with the PHD was associated with a 25% decreased mortality risk [7]. In a recent study, Landry et al. [9] showed beneficial cardiometabolic effects of plant-based vs omnivorous diets in identical twins. Springmann et al. [8] analyzed the health and environmental impacts of different diet scenarios for selected regions in the world and showed that in high-income countries, the change to more plant-based diets, as recommended by the PHD, would lead to improved nutrient levels, a reduced risk of premature death, and the reduction of environmental impacts.

While the evidence supporting the benefits of following the recommendations of the PHD is increasing, the actual food consumption of the global population still falls short of these guidelines [7,10,11,12,13]. This raises the question of how the dietary habits of the world’s population can be aligned with the recommendations of the PHD. Recently, Jochem et al. [14] described a new conceptual model for planetary health literacy that includes diet as an important dimension of planetary health [14], as food is related to all sustainable development goals [15]. In this model, the following definition of planetary health literacy is proposed:

“Planetary health literacy can be defined as the knowledge and competencies of accessing, understanding, appraising, and applying information in order to make judgements and take decisions regarding planetary health, across societies and for health-promoting, sustainable, and transformative actions.

Planetary health literate individuals and societies are enabled to sustain and promote their own health, population health, and the planet’s health. They are able to adopt a more holistic understanding of their health embedded in natural systems they are living in. Based on their knowledge and attitude, they take decisions that reflect and foster the interconnectedness of human health and well-being with the state of the natural systems and related areas of nature-society interactions.” [14] (p. 5).

Universities play a significant role within the framework of education for sustainable development (ESD). University students, in their role as potential future experts and leaders in their fields, can serve as important change agents of society [16]. The role of universities is to train these aspiring experts and equip them with the necessary knowledge and literacy for a sustainable future [17]. By passing on sustainability- and health-related knowledge and skills in their future professional roles, students become agents of change [17], helping to promote sustainable behavior [18] and thereby contributing to social transformation. 

Despite the growing inclusion of topics related to climate change and sustainability in the different disciplines and degree programs, several studies analyzing the actual food intake of university students show that the average student diet differs largely from the recommendations of the PHD or national dietary guidelines, as they are, on average, low in fruits, vegetables [19,20], and whole grains [19], and they frequently show a high consumption of meat and meat products [21,22], even though the body of literature postulating a moderate or declining meat consumption among university students is increasing [19,23]. Apart from situational factors such as time constraints [24,25,26,27] and financial constraints [25,28], the reasons for unhealthy and unsustainable dietary habits among students appear to include missing or misleading information about the healthiness and/or sustainability of foods [24,29] and a lack of culinary competencies [28]. At the same time, improvements in diet-related knowledge, increased cooking skills, and a higher frequency of cooking seem to be facilitators for healthy and sustainable diets [26,30].

Teaching kitchens have great potential to overcome these barriers and to translate nutrition education (and the planetary health diet) into the everyday lives of participants [31]. An increasing number of studies demonstrate the high effectiveness of teaching kitchens in enhancing culinary skills, improving dietary habits and lifestyles of participants, as well as increasing the nutritional and dietetic counseling competency of health professionals [32,33,34]. While there have been multiple studies analyzing the effects of teaching kitchen interventions on university students, none of these have examined the extent to which teaching kitchen courses can enhance the nutrition-related planetary health literacy of students to act as future multipliers and change agents to pass on nutrition- and sustainability-related knowledge and skills. This is why we developed a teaching kitchen-based PHD curriculum, built upon the methodological groundwork established by Neumann [35], that aims at translating nutrition- and sustainability-related knowledge and skills into the everyday lives of university students to equip them with the necessary competencies to act as change agents for a sustainable future in the professional positions they will occupy after their academic training [36].

In this paper, we pursue three objectives. These are:

1. To introduce the teaching kitchen-based PHD curriculum and to explain the theoretical framework in which it is embedded.
2. To assess the practical feasibility of the course concept in higher education with university students as the target group.
3. To examine whether the PHD curriculum has an effect on the ability of university students of all degree programs to advise individuals in their professional or personal environment regarding a more sustainable or healthier diet, referred to as planetary health diet literacy.

## 2. Materials and Methods

### 2.1. Design

This paper is the result of an (ongoing) study that is designed as a pre–post-intervention and is being conducted at the University of Göttingen, Germany. The intervention consists of a seven-week PHD curriculum in a teaching kitchen setting. Since the curriculum addresses university students of all fields of study and is not aimed at specific degree programs, it is offered via the platform ZESS (Zentrale Einrichtung für Sprachen und Schlüsselqualifikationen der Universität Göttingen, Central Institution for Language and Key Qualifications of the University of Göttingen), which is accessible to all students at the University of Göttingen. 

The feasibility of the PHD curriculum with university students as the target group is assessed on the basis of the evaluation of an official and course-independent teaching evaluation conducted by ZESS. The intervention’s impact on planetary health diet literacy is assessed using a customized digital pre- and post-survey completed by the course participants before the first session and after the last session. This survey is part of an anonymized teaching evaluation tailored to the course. 

### 2.2. Participants

The PHD curriculum is an elective class at the University of Göttingen. Students from all disciplines, semesters, and degree programs that are enrolled at the University of Göttingen can register for the PHD curriculum through the ZESS platform of the University of Göttingen. Prior to the course, students have the opportunity to inform themselves about the content of the course through the module description (see Supplementary Materials). The allocation of course slots is based on the order of registration. 

During the pilot phase from April 2022 to September 2022, a total of three courses were conducted. For courses 1 and 2, there were 12 slots available for each course. Due to limited space, only 6 slots were available for the third course. Eleven students registered for the first course. Out of these, ten students attended the first day of the course. One student dropped out, as they could not fit the course into their schedule. Nine students successfully completed the course. Twelve students registered for and successfully completed the second course. For the third course, six students initially registered. Five students attended and completed the course successfully. Overall, 26 students successfully completed one of the three PHD courses during the pilot phase. 

During the first session of each course, the research activities were explained to the participants. All the participants included in the pilot study signed the consent form before they provided any data. The ethics committee of the UMG, University Medical Centre, has no objections to the anonymized publication of the teaching evaluation. 

### 2.3. The Planetary Health Diet Curriculum

The PHD curriculum consists of seven sessions, each lasting three hours, held at weekly intervals. Each session starts with a one-hour seminar, followed by two hours of hands-on cooking in the teaching kitchen. The one-hour seminar is based on the pedagogical concept of a flipped classroom, which is associated with positive effects on students’ learning performance [37], by promoting autonomy and competency through the inversion of the roles of instructor and students [38]. For this purpose, the students work on the key topics of the course (see Table 1) at home and elucidate them to their peers through presentations during the seminar. Basic literature is made available to the students for the preparation of the presentations, but the students are also encouraged to incorporate literature from their own research. Each session starts with two student presentations. The presentations are held from single students or in groups of two students maximum. The presenters are encouraged to prepare discussion questions that will be discussed with their fellow students following the presentation.

The selection of key topics for the seminar is based on the concept of planetary health literacy. According to Jochem et al. [14], planetary health-literate individuals are capable of improving their own health, population health, and planetary health through a holistic understanding of how their health is embedded in the natural system they live in [14]. Accordingly, the topics relate to individual health (e.g., (1), (2), (6), (7), (8), (10), (14)), the health of the (global) population (e.g., (1), (2), (3), (4), (5), (6), (12)), and planetary health (e.g., (1), (3), (4), (5), (8), (9), (10), (11), (13), (14)), with connections between these different targets being established and discussed within all topics. 

The seminar is followed by a two-hour hands-on cooking class, during which the students prepare recipes in groups. These are favorite recipes submitted by the students themselves before the start of the first day of the course. In the teaching kitchen, the students prepare the recipes in two versions. The original version is cooked, and a variation/reformulation of the recipe is also prepared, where ingredients are exchanged with the aim of making the dish more sustainable, healthier, or both. Changes to the recipe could include, for example, substituting an animal protein source with a plant-based one, using whole wheat flour instead of white flour, increasing the proportion of vegetables, or opting for a fat with a nutritionally favorable fatty acid profile. During the cooking class, the students also learn about nutrient- and energy-saving preparation methods and how to modify ingredients to reduce the carbon footprint of dishes or to increase nutrient density. They practice the use of peel waste as a basis for vegetable broth or as a recipe ingredient, as well as correct storage methods in the refrigerator or pantry. During every session, the students calculate the carbon footprint of all the prepared recipe variants using the web-based application KlimaTeller (climate plate), a joint project of NAHhaft e.V. and Greentable e.V. in cooperation with Eaternity [39]. 

At the end of the cooking class, the prepared dishes are tasted during a communal meal. The recipe variants are compared and discussed regarding potential challenges during preparation, their nutritional value, their carbon footprint, and their taste. In addition to these aspects, the focus of the dish evaluation is particularly on enjoyment. The result of the tasting can thus also be that the original version of the dish tastes better than the variation, and one may choose to continue enjoying the dish in its original form, albeit less frequently, considering potential adverse effects on the climate and/or health.

The examination consists of a seminar presentation and the submission of a scientific poster on the presentation topic by the end of the course.

The course is conducted by an interprofessional team consisting of a nutritionist, chefs specially trained in nutrition and dietetics, a sustainability educator, a social scientist and pedagogue, and a physician. 

### 2.4. Theoretical Framework 

The PHD curriculum is based on the theoretical framework of social–cognitive theory (SCT). SCT was first proposed by Albert Bandura and examines the factors influencing human behavior and the processes of learning [40]. It offers a renowned approach for better understanding dietary behavior change [41]. In consequence, the theory is widely used in nutrition education and public health strategies [40,42], or as a theoretical framework for dietary interventions [33,43,44,45]. The key constructs of SCT include, among others, outcome expectations, self-efficacy, observational learning, reinforcement, and facilitation [40]. 

Outcome expectations relate to the assumption of the individual about the results of their actions. Due to the human capability for anticipatory thinking, the consequences of one’s actions in the future can also influence and motivate one’s present behavior [46]. However, this presupposes that the learner is aware of the consequences of his or her actions. A central problem of sustainability-related impacts is that the ecological and societal consequences of dietary behavior are usually not experienced in the form of an immediate effect [47]. This is due to the fact that the consequences often manifest themselves in the context of global problems, which predominantly occur on other continents and are also delayed in time. In addition, the effects are often not recognizable to consumers as a direct consequence of their consumption behavior. This makes it difficult for consumers to understand the causal linkage between their actions and (global) consequences. This missing immediate effect of sustainability-related consequences of dietary habits can also be extrapolated to the health implications of one’s behavior. For instance, a cancer diagnosis resulting from years of unfavorable dietary habits may not be perceived as a direct consequence of one’s eating habits. To form outcome expectations about the consequences of individual and global food consumption and about the interconnectedness of individual dietary decisions and planetary health, the one-hour seminar provides corresponding information embedded in 14 key topics, as shown in Table 1. 

While knowledge and outcome expectations are prerequisites for behavior change, self-efficacy beliefs, which are the individual conviction of one’s ability to perform a desired action [40], are essential to initiate changes in behavior [48]. Active participation in the cooking process strengthens students’ belief in their ability to implement sustainable cooking practices. They experience themselves as capable of following a recipe and modifying it to create a more sustainable or healthier version of the dish.

Observational learning enables the individual to acquire integrated behavior patterns without having to build them on their own through trial and error [46]. During the two-hour cooking classes, the instructors and fellow students serve as models that facilitate observational learning and immersive learning following cognitive apprenticeship and situated learning theories [49,50]. 

The discussions in the plenum after the presentations and the shared meal serve as social reinforcement. Through the exchange of thoughts and opinions, students can solidify their views and deepen their understanding. The communal evaluation and comparison of the dishes provide another form of reinforcement.

Finally, facilitation, as one of the fundamental approaches that describes environmental influence on behavior, plays an important role in the adaptation of a new behavior. Facilitation makes the behavior easier to perform by offering new and relevant structures or resources [48]. This means that barriers to the promoted behavior must be identified and options to remove or overcome these barriers should be introduced or developed [40]. As time constraints [24,25,26], financial constraints [25,28], missing or misleading information about the healthiness and/or sustainability of foods [24,29], and a lack of culinary competencies [28] have been identified as main barriers to a healthier and more sustainable diet for the target group, necessary knowledge and skills to overcome these barriers are provided and practiced during the course, and the students are supported to develop strategies to achieve a sustainable and balanced diet, even with time constraints and limited financial resources. 

### 2.5. Instruments

The feasibility was tested via the responses to corresponding questions of the official and course-independent teaching evaluation of the ZESS, in which the students anonymously and voluntarily rated their overall satisfaction with the course, their learning gain, and the level of course requirements. The items that were evaluated for this purpose were “In an overall assessment, I rate the course as …”, “The overall level of demand for the course is …”, and “I learn a lot in this course”. The students responded to the teaching evaluation of the ZESS during the seminar on the second-to-last day of the course. 

To investigate the impact of participating in the PHD curriculum on self-assessed planetary health diet literacy, we developed a 14-item survey adapted from Razavi et al.’s survey [34]. The authors examined the impact of indication-related cooking courses for medical students on their self-assessment of patient counseling competencies across 25 nutritional topics using a 3-point Likert scale. The students were asked about their confidence in advising patients on various medical nutrition-related indications [34]. For the present study, the opening question was revised to “I am confident that I can explain the following topics to another person (e.g., boyfriend/girlfriend, colleague)”. The scale items each reflected a core aspect of the key topics in the PHD curriculum (see Table 1), representing literacy in various nutrition-related aspects of individual, public, or planetary health. Instead of using the 3-point Likert scale, a 7-point Likert scale was employed to obtain more precise information about the differences in the pre- and post-intervention. 

The participants completed pre- and posttest assessments in the teaching kitchen in the presence of the course instructors to avoid the use of assistance tools. The students responded to the pre-survey at baseline, directly before the start of the first session, and to post-survey after the last session. The survey was completed anonymously and on a voluntary basis. To link the pre- and post-data, the students were asked to follow instructions to create an individual code that did not allow for any conclusions about one’s own person to preserve anonymity.

### 2.6. Data Collection and Analysis

The teaching evaluation of the ZESS was conducted via Stud.IP (Studienbegleitender Internetsupport von Präsenzlehre, online-support for in-person learning throughout the study). The analysis of the response frequencies was conducted automatically by the system and made available for download to the instructors of the PHD curriculum upon completion of the course. 

The data collection was conducted using the free, web-based application LimeSurvey (Version 3.24.2 + 201020). Subsequently, the data were transferred to Microsoft Excel Software systems (Version 16.75.2) for data clearance. All datasets for which no corresponding pre- or post-dataset could be identified were excluded. The reasons for exclusion were non-compliance with the pseudonym creation instructions, resulting in a non-reproducible pseudonym, or premature course withdrawal. The statistical analysis was performed using IBM SPSS Statistics (29.0.1.0). 

To compare the pre- and post-survey results, we calculated the percentage change by determining the mean difference in the 14 categories of planetary health diet literacy. We also conducted a post hoc test for significance. The normal distribution of the data was tested using Q–Q plots. The results of the pre- and post-intervention survey were compared using paired *t*-tests with *p*-values < 0.05. 

## 3. Results

### 3.1. Pilot Cohort

During the pilot phase from April 2022 to September 2022, 35 students enrolled in the PHD curriculum. Of these students, 29 attended the first session, 27 students met the attendance requirement of 80%, and 26 students fulfilled the required examination performance. These 26 students constitute the final study sample. 

Table 2 shows the sample characteristics of the pilot cohort. Out of the 26 course graduates, 57.7% (*n* = 15) of the participants were female. The mean age at the time of course participation was 24.4 years. The youngest participant was 20 years old, and the oldest participant was 33 years old. A total of 57.7% of the participants were enrolled in a bachelor’s degree program, and 42.3% were enrolled in a master’s degree program. None of the participants were enrolled in a Ph.D. program. 

### 3.2. Feasibility of the PHD Curriculum

The feasibility was tested via the results of the teaching evaluation of the ZESS. A total of 24 students provided responses to the questions “In an overall assessment, I rate the course as …” and “The overall level of demand for the course is …”. The statement “I learn a lot in this course” was assessed by 25 students. 

**Figure 1:**
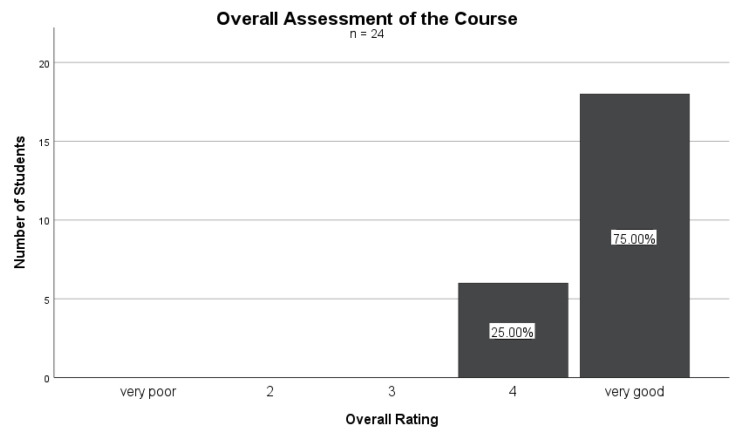
Overall assessment of the PHD curriculum (*n* = 24).

As shown in Figure 1, on a scale from 1 to 5, where 1 represents “very poor” and 5 represents “very good”, 75% (*n* = 18) of the students rated the course with a 5 in the overall evaluation. A total of 25% rated the course with a 4. The other ratings were not occupied. The mean rating was 4.75 (SD = 0.442). The fact that 75% of the students gave the course the highest rating indicates that the course was overall very well-received. The remaining 25% rated the course with a 4, which is also a positive evaluation. The fact that no lower rating levels were given suggests that all of the (responding) students seemed to have been satisfied with the course.

**Figure 2:**
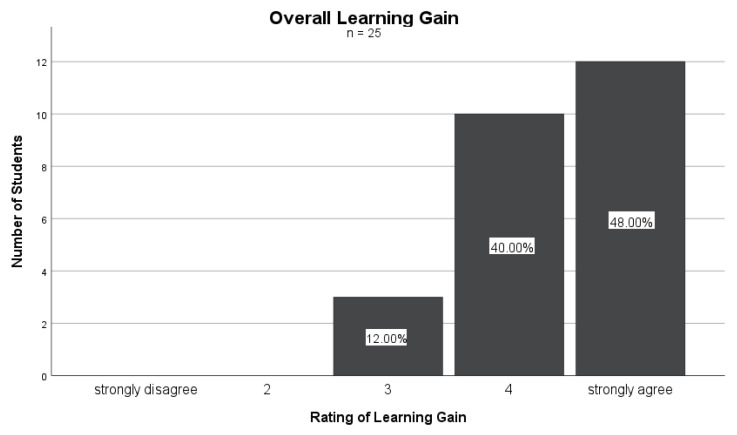
Overall learning gain through the PHD curriculum (*n* = 25).

Figure 2 shows how the students rated their learning gain through the PHD curriculum. The extent of learning gain was assessed on a Likert scale from 1 (“strongly disagree”) to 5 (“strongly agree”). In response, 48% (*n* = 12) of the students chose 5, 40% (*n* = 10) chose 4, and 12% (*n* = 3) chose 3, with a mean of 4.36 (SD = 0.700). This result suggests that a significant portion of the students (88%) perceived a substantial learning gain, providing the highest rating of 5 or 4 on the Likert scale. 

**Figure 3:**
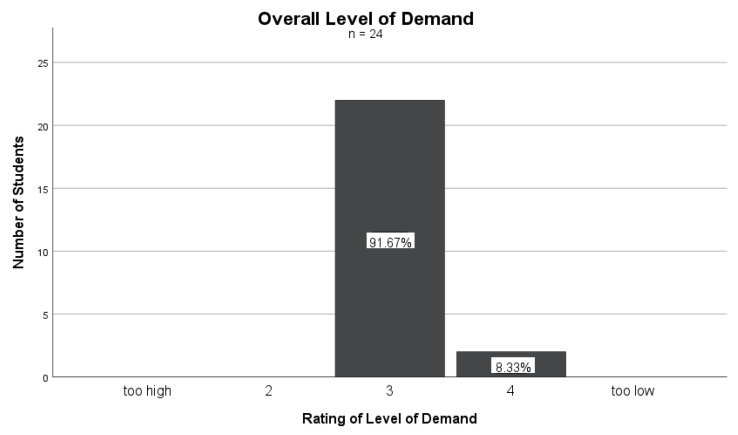
Overall level of demand of the PHD curriculum (*n* = 24).

The level of demand for the course was assessed on a scale from 1 (“too high”) to 5 (“too low”) and is shown in Figure 3. A total of 92% of the students rated it as a 3, and 8% rated it as a 4. The mean rating was 3.08 (SD = 0.282). The result indicates that most of the respondents perceived the level of demand for the course as just right. Only 8% would have preferred a slightly higher level. Therefore, the difficulty level was appropriate for the majority of the students.

In conclusion, our results suggest that the PHD curriculum is feasible with university students as the target group, since the course appears to have been very well-received by the majority of students, providing substantial learning growth and maintaining a suitable level of demand.

### 3.3. Changes in Planetary Health Diet Literacy

In total, the pre-test and post-test datasets of the survey could be successfully matched for 26 individuals. Table 3 shows the mean changes in self-assessed planetary health diet literacy on the 14 seminar topic-related categories. The PHD curriculum had positive effects in all the categories. 

The largest percentage change was observed with an increase of 97.63% for the category “planetary health diet”, followed by “food-based dietary guidelines of the DGE” at 71.43%. The smallest change occurred for the categories “consequences of global livestock farming” (20.46%) and “plant-based protein sources” (20.74%). 

The significant increase in confidence in explaining the PHD can be explained by the fact that a large portion of the students, when asked during the course, reported never having heard of the PHD before the course. This observation from our course is supported by findings of Klünder et al. [51], who found that 74% of students enrolled in health-related programs in Bavaria, Germany, had not even heard of planetary health in general. On the same note, the literature shows that the food-based dietary guidelines of the German Nutrition Society (DGE) are only known by 14% of adults, with an even lower awareness level among younger respondents [52], thus explaining our result. 

The participants’ high initial literacy regarding the consequences of global livestock farming and plant-based protein sources is likely due to the regular public discourse on the consequences of meat consumption and the substitution of animal protein with plant-based protein. 

The post hoc paired *t*-tests showed that the calculated differences in planetary health diet literacy between the pre- and post-intervention were significant for all 14 categories. Despite variations in the magnitude of the percentage changes across the categories, the results demonstrate that the PHD curriculum effectively promoted the students’ planetary health diet literacy in all 14 categories. This indicates the curriculum’s strong potential to empower university students to become planetary health diet-literate individuals. 

## 4. Discussion

We conducted this pilot study using university students from the University of Göttingen, Germany as representatives of other national and international university students to test the feasibility of a PHD curriculum in a teaching kitchen setting as a teaching offering in higher education and to analyze its effects on university students’ self-assessed planetary health diet literacy. 

The results of the official and independent teaching evaluation of the ZESS indicate that the PHD curriculum is feasible in higher education with students of diverse academic backgrounds, given the 100% positive overall rating, the 88% agreement on a high learning effect, and the 92% agreement that the level of demand of the curriculum is ideal. 

Considering the fact that the participating students came from various study programs and different degree programs, achieving a high learning effect and ensuring a level of demand suitable for all participants were crucial prerequisites for the success of the PHD curriculum. As participants bring varying levels of prior knowledge about nutrition and sustainability, it must be ensured that the theoretical content conveyed in the seminar is understandable for everyone while simultaneously meeting the standards of academic communication. The results of the ZESS teaching evaluation indicate that this has been well-achieved in our curriculum. This may be attributed to the fact that the students were responsible for conveying the key topics of the seminar to their peers through presentations, as well as through the associated role reversal of teachers and learners, following the concept of the flipped classroom. This consideration is supported by previous study results indicating that the performance of students with diverse backgrounds can benefit from the application of the flipped classroom concept [53,54].

The results of the pre–post-survey analysis indicate that the PHD curriculum has the potential to significantly increase the planetary health diet literacy of university students. One possible explanation for this result might be that the PHD curriculum addresses all four core competencies deemed necessary for the education of planetary health-literate individuals, according to Jochem et al. [14], which are:

1. Access/obtain information regarding planetary health.
2. Understand information regarding planetary health.
3. Appraise/judge information regarding planetary health.
4. Apply/use information relevant to planetary health.

Table S2 (Supplementary Materials) shows examples of key topics of the PHD curriculum and of their application regarding these four core competencies of planetary health literacy. 

Participants in the curriculum develop the ability to access information about the interconnection of individual health and actions, and planetary health (first core competency) through research for their presentations on the key topics of the seminar. In addition to the provided literature, students are encouraged to use scientific literature from their own research. This helps them distinguish between scholarly sources and potentially unreliable information on the internet. 

The ability to understand the assessed information about the interconnection of individual health, actions, and planetary health (second core competency) is achieved by addressing comprehension questions following the presentations. Additionally, the plenary discussions after the presentations contribute to a deeper understanding of the presented content. 

The course also facilitates the development of the ability to interpret and evaluate assessed and understood information and to weigh on its basis the pros and cons of consuming a certain food or food group against each other (third core competency) through plenary discussions. Here, different disciplines, opinions, and values intersect, leading to the articulation of pros and cons regarding the consumption or handling of food, from which students can draw conclusions. The evaluation and consideration of various pros and cons also take place through the tasting of recipe variations during the communal meal. This process also allows for the consideration of taste in the decision-making. 

For acquiring the ability to make an informed decision regarding human health and/or activities embedded in natural systems (fourth core competency), the teaching kitchen setting in the PHD curriculum plays a crucial role. Various sources have shown that culinary skills are of paramount importance for the adoption of healthier and more sustainable dietary habits among students [23,26,30]. This means that the mere realization that the disadvantages of certain dietary behaviors outweigh the benefits for human or planetary health is not sufficient to change this behavior. It must also be conveyed how alternative behaviors can be implemented. In the PHD curriculum, this is accomplished through hands-on cooking experiences in the teaching kitchen. Students practice modifying their own familiar recipes from home to create healthier and more sustainable dishes. This reduces the threshold for repeating these actions at home. The translation of acquired competencies into everyday life and actions is further facilitated by taking into account situational conditions and potential barriers in the study, such as a limited budget [28] and time constraints [24,25,26], when planning and creating recipe variations.

Previous studies have already demonstrated the high effectiveness of teaching kitchen-based university courses in enhancing students’ abilities to impart acquired nutritional knowledge and skills to others [34,55,56,57]. This suggests that the teaching kitchen setting in the present study may have significantly contributed to improving the students’ planetary health diet literacy. These findings are primarily derived from studies that investigated the effects of medical school courses in teaching kitchen settings on the self-assessed competency or confidence of medical students to counsel their future patients on dietary changes to treat and prevent diet-related diseases. To the best of our knowledge, this is the first study to investigate the effects of a teaching kitchen-based curriculum that focuses not only on healthy but also sustainable nutrition on the ability of university students from various study and degree programs to advise on sustainable and healthy diets, here referred to as planetary health diet literacy, in their professional or personal environments.

The goal of the PHD curriculum is not only to empower university students to make planetary health-literate decisions about their individual food choices, but also to equip them to make informed decisions about planetary health in their future professional role, which may include decision-making positions in politics, business, and society. While individual dietary choices, especially when made collectively, can have an impact on the food environment from a bottom-up approach, the decisions of individuals in influential positions can multiply this effect manifold and significantly influence public health, global health, and planetary health [14,58]. It can also be assumed that individuals with a strong understanding of planetary health literacy in the field of nutrition are likely to make more sustainable decisions in non-nutrition-related areas, as previous study results suggest that individuals who adopt sustainable dietary practices also tend to make more sustainable decisions in other aspects of their lives [59].

In this context, it is essential for students in health-related disciplines to have knowledge and literacy about the interconnections of human and planetary health, as well as students from all disciplines [51,60,61]. Only in this way can we ensure that future leaders and decision-makers, including political influencers, many of whom have not received health-related training, are equipped to become individuals who are literate in planetary health. This will enable them to make informed decisions, thereby contributing to shaping a sustainable future [60].

Our study has different limitations. As we measured the effects of the PHD curriculum on planetary health diet literacy by comparing baseline data to post-intervention data that was collected directly after the last session, we can only draw conclusions about the short-term impact of the course. To allow statements about the long-term effects, the collection of follow-up data is planned. Participants in the course will be contacted again six months after the last session and asked to respond to the survey.

The significance of mean changes in planetary health diet literacy was only tested post hoc due to the limited sample size of 26 of the pilot cohort. Consequently, further analyses with larger samples are necessary to substantiate and confirm the indications found here regarding the potential of the PHD curriculum.

Furthermore, there is a selection bias in our study sample, as the students actively enrolled in the PHD curriculum, knowing that the course was about sustainable and healthy nutrition, and that hands-on cooking would be part of the course. In consequence, it can be assumed that primarily students who already had a certain interest in cooking and in healthy, sustainable nutrition enrolled. Due to this interest, the participants probably brought a relatively higher level of prior knowledge about nutrition- and sustainability-related topics than the average population. The fact that the course was still able to increase the planetary health diet literacy of the participants suggests that the effects could be even greater for less-interested students or other population groups.

For this pilot study, only a short survey with some sociodemographic questions and questions used to measure planetary health diet literacy was used. However, the scale we used to measure planetary health diet literacy solely focuses on the students’ ability to explain various core topics related to individual, public, and planetary health to others, thus only assuming the first and second core competencies of planetary health literacy outlined by Jochem et al. [14]. These competencies relate to the ability to acquire and understand relevant information. By assessing changes in food choices in future surveys, insights into the third and fourth core competencies could be drawn. According to these competencies, planetary health-literate individuals are capable of evaluating and weighing relevant information, and based on this, making informed (nutrition) decisions, suggesting, for example, a higher consumption of plant-based foods and a lower consumption of animal-based foods. In the further implementation of this course, a longer questionnaire will therefore be used, allowing for insights into these additional aspects.

The PHD curriculum, which has been very well-received by university students in Göttingen, Germany, can easily be implemented and replicated at other national and international universities. Apart from an equipped teaching kitchen, the course requires minimal course materials, as students actively shape a significant portion of it through their presentations and the submission of recipes. The literature provided to the students for their presentations is largely international. Individual papers or reports that pertain to Germany or are in German can be easily replaced with minimal effort. Through replication at other universities, the PHD curriculum could empower students worldwide to become replicators and promoters of healthy and sustainable dietary practices, thus supporting the urgently needed transformation of food systems.

Further multicenter research with larger sample sizes, an adapted survey, and long-term follow-up is warranted to substantiate the preliminary findings and assess the long-term sustainability of increases in planetary health diet literacy observed in this pilot study. 

## 5. Conclusions

The PHD curriculum is a teaching kitchen-based course offered at the University of Göttingen, Germany, that addresses university students across all disciplines and degree programs. The curriculum integrates knowledge transfer using the inverted classroom concept with hands-on cooking in the teaching kitchen to translate theoretical content into practical, real-world skills. In this pilot study, we introduced the concept of the PHD curriculum and showed its feasibility based on a pilot cohort of students of various academic backgrounds. The survey results show that the PHD curriculum improved the planetary health diet literacy of the participants, empowering the participants with the potential to make informed decisions for the benefit of planetary health in future professional positions. Further studies with larger sample sizes are needed to confirm these results. The implementation of comparable curriculums at universities worldwide would be desirable to harness the potential of students as change agents for more sustainable food systems.

## Figures and Tables

**Table 1:** Key topics addressed during the seminar.

Session	Key Topic
1	(1) The 10 guidelines of the German Nutrition Society (DGE) (2) Characteristics of the Mediterranean and vegetarian diet
2	(3) Definition, dimensions, and goals of sustainable nutrition (4) The planetary health diet
3	(5) The planetary health diet—comparison and criticism (6) Less is more—meat consumption from a sustainability perspective
4	(7) Sustainably nurtured—animal- vs. plant-based foods (8) Long-term suitability of dietary patterns
5	(9) Vegan diets (10) Front-of-package food labels and what they imply
6	(11) Are organic foods always the better choice? (12) Global food security—(how) can we feed 10 billion people?
7	(13) Food waste—extent and prevention strategies (14) Sustainable nutrition with restricted time and budget

**Table 2:** Sample characteristics of the pilot cohort (*n* = 26).

Characteristic	
Gender	
Female (*n* (%))	15 (57.7)
Male (*n* (%))	11 (42.3)
Diverse (*n* (%))	0 (0)
Mean age (years ± SD)	24.4 (±2.80)
Degree program	
Bachelor’s (*n* (%))	15 (57.7)
Master’s (*n* (%))	11 (42.3)
Ph.D. (*n* (%))	0 (0)

**Table 3:** Mean changes in planetary health diet literacy.

Category	Mean (SD) Pre	Mean (SD) Post	MD (SD) Pre-Post	MD (%) Pre-Post
Food-based dietary guidelines of the DGE	1.62 (0.94)	3.42 (1.03)	1.81 (1.27) *	71.43
Mediterranean diet	2.04 (0.92)	3.38 (1.02)	1.35 (1.09) *	49.44
Dimensions of sustainable nutrition	2.77 (1.12)	4.27 (0.78)	1.50 (1.14) *	42.61
Planetary health diet	1.19 (0.40)	3.46 (1.07)	2.27 (1.08) *	97.63
Consequences of global livestock farming	3.73 (1.15)	4.58 (0.58)	0.85 (1.16) *	20.46
Plant-based protein sources	3.50 (1.11)	4.31 (0.62)	0.81 (0.98) *	20.74
Long-term suitability of dietary patterns	2.65 (1.26)	4.12 (0.86)	1.46 (1.33) *	43.43
Vegan diets	1.73 (0.87)	3.12 (1.28)	1.38 (1.24) *	57.32
Food labels	2.23 (1.03)	4.08 (0.80)	1.85 (1.12) *	58.64
Advantages and disadvantages of organic foods	3.00 (1.06)	4.15 (0.73)	1.15 (1.26) *	32.17
Greenhouse gas emissions of different foods	3.27 (1.22)	4.27 (0.67)	1.00 (1.23) *	26.53
Global food security	2.35 (1.16)	3.85 (0.88)	1.50 (1.14) *	48.39
Food waste	3.38 (0.85)	4.42 (0.70)	1.04 (0.92) *	26.67
Highly processed foods	2.85 (1.16)	4.12 (0.82)	1.27 (1.19) *	36.44
Overall	2.60 (0.60)	3.97 (0.52)	1.37 (0.69) *	41.70

SD = Standard deviation, MD = Mean difference; * *p* < 0.001.

## Data Availability

The data presented in this study are available from the corresponding authors upon request. The data are not publicly available due to privacy restrictions.

## Supplementary Materials

The following supporting information can be downloaded at: https://www.mdpi.com/article/10.3390/nu16040521/s1, Table S1: Planetary health diet literacy paired *t*-test; Table S2: Examples of key topics of the PHD curriculum and of their application regarding the four core competencies of planetary health literacy; module description of the PHD curriculum; pre- and post- survey.
