# Supplementary material for: University Students as Change Agents for Health and Sustainability: A Pilot Study on the Effects of a Teaching Kitchen-Based Planetary Health Diet Curriculum
Source: Nutrients. 2024 Feb 13;16(4):521. doi: 10.3390/nu16040521 (PMC10892560; doi:10.3390/nu16040521)
Supplement: Supplementary file 1 [file nutrients-16-00521-s001.zip › nutrients-2826801-supplementary.pdf]

Supplementary Materials

Table S1. Planetary health diet literacy paired *t*-test.

Category	T-value (*t*)	Degrees of freedom (*df*)	Significance (*p*) (1-Tailed)	Cohen's *d*
Food-based dietary guidelines of the DGE	-7.284	25	<0.001	1.266
Mediterranean diet	-6.278	25	<0.001	1.093
Dimensions of sustainable nutrition	-6.708	25	<0.001	1.140
Planetary health diet	-10.722	25	<0.001	1.079
Consequences of global livestock farming	-3.734	25	<0.001	1.156
Plant-based protein sources	-4.200	25	<0.001	0.981
Long-term suitability of dietary patterns	-5.588	25	<0.001	1.334
Vegan diets	-5.715	25	<0.001	1.235
Food labels	-8.402	25	<0.001	1.120
Advantages and disadvantages of organic foods	-4.688	25	<0.001	1.255
Greenhouse gas emissions of different foods	-4.136	25	<0.001	1.233
Global food security	-6.708	25	<0.001	1.140
Food waste	-5.783	25	<0.001	0.916
Highly processed foods	-5.461	25	<0.001	1.185
Overall	-10.121	25	<0.001	0.69203

Table S2. Examples of key topics of the PHD curriculum and of their application regarding the four core competencies of planetary health literacy.

	**Access/obtain information regarding planetary health**	**Understand information regarding planetary health**	**Appraise/judge information regarding planetary health**	**Apply/use information relevant to planetary health**
Overarching contents regarding planetary health literacy from Jochem et al. [14] (p. 7)	*Ability to access information on the interconnectedness of human health, the state of the natural systems, and human activities for planetary health.*	*Ability to understand information on the interconnectedness between human health, human activities, and the state of the natural systems.*	*Ability to interpret and evaluate information on the diverse relationships between human activities, human health, and the state of the natural systems.*	*Ability to make informed decisions regarding human activities or human health in the context of the state of the natural systems.*
Example from Jochem et al. [14] (p. 7) Healthy and sustainable diet at the individual level	*I am able to access information that informs me on nutritional value (calories, vitamins etc.) and impact on ecosystem health (e.g. emissions because of transportation, waste of natural resources because of crop growing, etc.).*	*I am able to easily understand the information provided and I can derive the meaning of this information regarding the benefits for my own health, ecosystem health by choosing certain fruit or vegetables, and dismissing others.*	*I am able to interpret the information provided. It enables me to evaluate the information and helps to weigh the pro and cons and thus decide which vegetable or fruit to buy.*	*I am able to make an informed decision and draw a planetary health literate decision regarding which fruit or vegetable to buy.*
Example 1 from the planetary health diet curriculum, topic (6) "Less is more – Meat consumption from a sustainability perspective": Healthy and sustainable diet at the individual level	I am able to access information on the nutritional value and benefits of steak consumption, but also on the potential health risks associated with high consumption of red meat. At the same time, I can access information on ecosystem impacts of meat consumption (e.g. deforestation of rainforest for feed cultivation, emissions (especially methane gas) etc.) and on animal welfare aspects.	I am able to understand which values the consumption has for my personal health (calories, protein, iron, vitamin B12 etc.) and what the purchase implies regarding planetary health.	I am able to appraise this information and weigh the benefits of eating steak regarding my personal preferences and health against the potential harm on my health and the harm on the ecosystem.	I am able to make an informed decision if I want to buy the steak, because I like to eat steak and it is beneficial for my health, and to choose a product whose production meets my criteria for animal welfare in breeding, or if I choose not to buy it, due to the harmful impact on my health and the ecosystem.
Example 2 from the planetary health diet curriculum, topic (7)	I am able to access information on the differences in macro- and micronutrients between animal- and	I am able to understand which values the consumption of animal or plant-based foods has for	I am able to appraise this information and weigh the benefits eating foods of animal origin regarding my	I am able to make an informed decision if I want to buy (certain) animal-based foods, because some might

"Sustainably nurtured – Animal- vs. plant-based foods": Healthy and sustainable diet at the individual level	plant-based foods and on the different benefits and drawbacks for my health and the environment that the consumption of animal or plant-based foods is associated with. At the same time, I can access information on how to replace animal products with plant-based alternatives without experiencing a deficit in nutrient intake.	my personal health and what the purchase implies regarding planetary health. At the same time, I am able to understand that animal and plant-based foods differ in terms of their micro and macronutrients, and I am aware of considerations when substituting plant-based for animal-based foods.	health against its potential harm for my health and its harm for the environment and the benefits eating plant-based foods regarding my health and planetary health.	be beneficial for my health or if I choose not to buy (certain) animal-based foods, due to the potential for my health and the harm for the environment. If I choose to replace animal products with plant-based alternatives, I am able to select foods and preparation methods that ensure I am adequately supplied with all necessary nutrients.
Example 3 from the planetary health diet curriculum, topic (10) "Are organic foods always the better choice?": Healthy and sustainable diet at the individual level	I am able to access information on the advantages of organic food consumption for my health (e.g. lower pesticide contamination) and for the environment (e.g. higher biodiversity, conservation of surface waters and groundwater) but also on the possible disadvantages for global food security and greenhouse gas-emissions due to larger yield gaps and increased foreign agricultural land use to compensate for shortfalls in domestic supply.	I am able to understand which values the consumption has for my personal health (lower pesticide contamination etc.) and what the purchase implies regarding planetary health.	I am able to appraise this information and weigh the benefits eating organic foods regarding my health and the environment against its disadvantages for global food security.	I am able to make an informed decision if I want to buy organic foods, because it is beneficial for my health or if I choose not to buy them, due to disadvantages for global food security and greenhouse gas-emissions, or if I buy only specific foods from organic farming.

**Georg-August-Universität Göttingen**<br>**Modulnummer**<br>**Bezeichnung** Planetary Health Diet – Seminar und praktische Übungen im Teaching Kitchen für eine nachhaltige und gesundheitsförderliche Ernährung<br>**Englische Bezeichnung** Planetary Health Diet – Teaching Kitchen Class for a sustainable and healthy Diet	**Credits**<br>**SWS**<br>*3 C / 2 SWS*
**Lernziele/Kompetenzen:**<br>Wie wir essen wirkt sich nicht nur auf die individuelle Gesundheit und das Wohlbefinden aus. Unsere Ernährung beeinflusst auch Ressourcenverbrauch, Kulturlandschaft, Klima und Gesellschaft. Wissenschaft und Politik stehen vor der drängenden Herausforderung, Maßnahmen zu ergreifen, die der ständig wachsenden Bevölkerung auch in Zukunft eine gesundheitsförderliche und sichere Ernährung innerhalb der planetaren Grenzen ermöglichen und soziale Ungleichheiten verringern. Weltweite Referenz für einen Lösungsvorschlag ist die Planetary Health Diet der EAT Lancet Commission (2019). Die Ernährungsempfehlungen der Deutschen Gesellschaft für Ernährung (DGE) sind in hohem Maße dazu konkordant (2022). Beide stellen die Grundlage für dieses Modul dar. Im Rahmen des Seminars wird ein wissenschaftlich fundiertes Verständnis für die Grundlagen einer sowohl nachhaltigen wie auch gesundheitsfördernden Ernährung innerhalb der planetaren Grenzen generiert. Möglichkeiten der Integration einer entsprechenden Ernährungsweise in den Alltag werden erarbeitet. Im Anschluss werden die theoretischen Inhalte von den Studierenden im Rahmen von praktischen Übungen im Teaching Kitchen (Kochkurs) direkt in konkretes Handeln umgesetzt und beim gemeinsamen Essen diskutiert. Rezepte werden variiert und die jeweiligen Auswirkungen auf Gesundheit (Culinary Medicine), Nachhaltigkeit aber auch Sensorik und Akzeptanz herausgearbeitet. So wird das Gelernte durch praktische Anwendung gefestigt und die Studierenden verbessern wichtige Kulturtechniken und Kompetenzen für die Nahrungszubereitung. Zudem befähigen diese Kompetenzen dazu, eine Multiplikatorenfunktion in der Gesellschaft für die Transformation in Richtung nachhaltiger Entwicklungsziele (SDGs) zu übernehmen.<br><br>Nach erfolgreicher Teilnahme sind die Studierenden in der Lage,<br>• die Bedeutung einzelner Nahrungsmittel für die persönliche Gesundheit und das Klima (CO_2-Äquivaltente) einzuordnen.<br>• unterschiedliche Ernährungsweisen hinsichtlich ihrer Auswirkung auf Gesundheits- und die Nachhaltigkeitsdimensionen zu diskutieren.<br>• die Eigenschaften von Lebensmitteln (z.B. Convenience-Grad, ökologische Erzeugung, Regionalität) differenziert zu bewerten.<br>• ihr persönliches Ernährungsverhalten hinsichtlich seiner Nachhaltigkeit zu bewerten und ggf. zu modifizieren.<br>• verschiedene Zubereitungsweisen und -techniken sicher anzuwenden.<br>• Rezepte umzusetzen und bei Bedarf selbstständig abzuwandeln.<br>• Nachhaltigkeitsdimensionen kritisch-konstruktiv gegeneinander abzuwägen.	**Arbeitsaufwand:**<br><br>Präsenzzeit:<br>*28 Stunden*<br>Selbststudium:<br>*62 Stunden*

<table>
<tr><td colspan="2">

Lehrveranstaltungen und Prüfungen:

1. Bezeichnung
Planetary Health Diet – Seminar und praktische Übungen im Teaching Kitchen für eine nachhaltige und gesundheitsförderliche Ernährung (*Seminar/Vorlesung)*
Inhalte*: DGE-Empfehlungen, SDGs, Planetary Health Diet, nachhaltiges Ernährungsverhalten*

2. Bezeichnung
Planetary Health Diet – Seminar und praktische Übungen im Teaching Kitchen für eine nachhaltige und gesundheitsförderliche Ernährung (*praktische Übungen/Kochkurs)*
Inhalte*: Praktische Anwendung der Seminarinhalte durch die Auswahl und Zubereitung passender Gerichte.*

Prüfungsvorleistung:
Regelmäßige und aktive Teilnahme an den Vorlesungen, Kochkursen und Diskussionsrunden

Prüfung: Prüfungsform, Prüfungsdauer, **unbenotet?**
Eine mündliche Leistung (Präsentation, ca. 20 min), Erstellung eines wissenschaftlichen Posters

</td><td>

SWS einzeln:

1 SWS
(14 LVS gesamt)

1 SWS
(14 LVS gesamt)

</td></tr>
<tr><td colspan="3">**Prüfungsanforderungen:** Die Teilnehmerinnen und Teilnehmer erbringen den Nachweis über die erworbenen Kompetenzen über eine Präsentation (mündlich, ca. 20 Minuten) und die Erstellung eines wissenschaftlichen Posters</td></tr>
<tr><td>**Zugangsvoraussetzungen:**
keine</td><td colspan="2">**Empfohlene Vorkenntnisse:**
keine</td></tr>
<tr><td>**Sprache:**
Deutsch</td><td colspan="2">**Modulverantwortliche[r]:**
PD Dr. med. Thomas Ellrott</td></tr>
<tr><td>**Angebotshäufigkeit:**
Zwei- bis viermal pro Semester</td><td colspan="2">**Dauer:**
1 Semester</td></tr>
<tr><td>**Wiederholbarkeit:**
zweimalig</td><td colspan="2">**Empfohlenes Fachsemester:**</td></tr>
<tr><td colspan="3">**Maximale Studierendenzahl:**
24</td></tr>
<tr><td colspan="3">**Bemerkungen:**
Die Präsenzzeit bei den Kursen im Semester ist 7 Termine à 4 LVS (immer montags 17-20 Uhr).</td></tr>
</table>

Teil A: Umfrageorganisation

Die folgenden Angaben dienen der späteren Auswertung nach Kohorten.

A1. Ihr persönlicher Code:

Bitte generieren Sie als erstes einen persönlichen 10-stelligen Code gemäß nachfolgendem Muster: Felder 1 und 2 sind für den Ort des Kurses. Felder 3 und 4 entsprechen jeweils dem zweiten Buchstaben Ihres Vornamens und Nachnamens, Felder 5 und 6 dem Tag ihres Geburtstages, Felder 7 und 8 den Initialen des Namens Ihrer Mutter. Die letzten beiden Felder 9 und 10 entsprechen den ersten beiden Buchstaben Ihres Geburtsortes.

Beispiel: Kurs in GÖttingen, Frieda von Dannen, *03.12.1996; Wilma von Dannen, München GÖ R A 0 3 W D M Ü

WICHTIG: Bitte bemühen Sie sich, den Code genau nach Anleitung zu erstellen, damit Sie zu allen Befragungszeitpunkten den gleichen Code erhalten (die Anleitung wird bei allen Befragungen bereitsgestellt). Andernfalls können wir die Daten nicht zusammenführen und Ihre wertvollen Angaben nicht verwenden.

A2. Veranstaltungsnummer:

Bitte geben Sie hier die 6-stellige Veranstaltungsnummer an.

A3. Erhebungszeitpunkt der Umfrage:

- ☐ Vorbefragung (vor Kursbeginn)
- ☐ Nachbefragung (nach Kursende)
- ☐ Follow-up-Befragung (6 Monate nach Kursende)

A4. Angabe des aktuellen Semesters

- ☐ Sommersemester 2022
- ☐ Wintersemester 2022/2023
- ☐ Sommersemester 2023
- ☐ Wintersemester 2023/2024
- ☐ Sommersemester 2024

Teil B: Kommunikation

Nun würden wir gerne wissen, inwiefern Sie sich vorstellen können, die im folgenden genannten Themen einem Freund oder einer Freundin zu erläutern.

B1. Ich bin zuversichtlich, einer anderen Person (z.B. Freund / Freundin, Kollegen / Kollegin) folgende Themen erläutern zu können...

	Stimme gar nicht zu	Stimme eher nicht zu	Teil / teils	Stimme eher zu	Stimme voll und ganz zu
Wie eine Ernährung nach den 10 Regeln der Deutschen Gesellschaft für Ernährung (DGE) aussehen kann	☐	☐	☐	☐	☐
Die Besonderheiten der Lebensmittelauswahl bei der mediterranen Ernährung	☐	☐	☐	☐	☐
Welche übergeordneten Zieldimensionen nachhaltige Ernährung beinhalten kann	☐	☐	☐	☐	☐
Die Besonderheiten der Lebensmittelauswahl und mengenbezogenen Empfehlungen der Planetary Health Diet	☐	☐	☐	☐	☐
Mit welchen ökologischen Auswirkungen die globale Nutztierhaltung verbunden ist	☐	☐	☐	☐	☐
Welche pflanzlichen Lebensmittel einen hohen Proteingehalt besitzen	☐	☐	☐	☐	☐
Ob sich ein bestimmter Ernährungsstil oder eine Diät als langfristige Ernährungsweise eignen	☐	☐	☐	☐	☐
Über welche Lebensmittel man den Calcium-Bedarf bei einer veganen Ernährungsweise decken kann	☐	☐	☐	☐	☐
Welche Siegel und Label auf Lebensmitteln geschützt sind und welche eher Marketingzwecken dienen	☐	☐	☐	☐	☐
Mit welchen Vor- und Nachteilen der Konsum von Bioprodukten verbunden ist	☐	☐	☐	☐	☐
Wie sich Eigenschaften wie ökologische Herstellung, Saisonalität und Regionalität auf den CO2-Fußabdruck von Lebensmitteln auswirken können	☐	☐	☐	☐	☐
Welche Wege und Maßnahmen zur Erreichung von globaler Ernährungssicherheit diskutiert werden	☐	☐	☐	☐	☐
Welche Möglichkeiten es gibt, um die eigenen Lebensmittelabfälle zu reduzieren	☐	☐	☐	☐	☐
Wie man auch mit wenig Zeit eine ausgewogene und nachhaltige Ernährung im Alltag realisieren kann	☐	☐	☐	☐	☐

Teil C: Fragen zu Ihrer Person

Zum Abschluss bitten wir Sie, noch einige Fragen zu Ihrer Person zu beantworten.

C1. Bitte geben Sie Ihr Geschlecht an.

- weiblich ☐
- männlich ☐
- divers ☐
- keine Angabe ☐

C2. Bitte geben Sie Ihr Geburtsjahr an. Z.B. 1999.

C3. Bitte geben Sie Ihre Studienrichtung an, z.B. Physik.

C4. Bitte geben Sie den gegenwärtig von Ihnen angestrebten Abschluss an.

Hier ist nicht gemeint, welchen Abschluss Sie zukünftig noch erreichen möchten, sondern auf welchen Abschluss der Studiengang, in dem Sie derzeit eingeschrieben sind, abzielt.

- Bachelor ☐
- Master ☐
- Staatsexamen ☐
- Promotion ☐
- Sonstiges, und zwar: ☐

Sonstiges, und zwar:

C5. Haben Sie vor dem Studium bereits eine berufliche Ausbildung abgeschlossen?

- Ja ☐
- Nein ☐

C6. Bitte geben Sie an, welche berufliche Ausbildung Sie vor dem Studium abgeschlossen haben.

Z.B. Bachelor Grundschullehramt

C7. Haben Sie vor Ihrem aktuellen Studium bereits ein anderes Studium abgeschlossen?

Hier ist nicht gemeint, ob Sie z.B. bereits einen zugehörigen Bachelor in der von Ihnen gegenwärtig belegten Studienrichtung absolviert haben, sondern ob Sie bereits ein anderes, fachfremdes Studium abgeschlossen haben.

- Ja ☐
- Nein ☐

C8. Bitte geben Sie an, welches Studium Sie vor Ihrem aktuellen Studium abgeschlossen haben.

Z.B. Bachelor Grundschullehramt

C9. Haben Sie bisher schon einmal an einem Kochkurs teilgenommen?

- Ja, in der Schule. ☐
- Ja, an der Universität. ☐
- Ja, außerhalb der Schule und der Universität. ☐

Nein, ich habe bisher an keinem Kochkurs teilgenommen. ☐

Sonstiges ☐

Sonstiges

C10. Zuletzt können Sie uns gerne noch ein Feedback geben:

Was hat Ihnen an dem Kurs gefallen?

C11. Was hat Ihnen nicht so gut an dem Kurs gefallen? Wo sehen Sie Verbesserungspotenzial?

Wir bedanken uns für Ihre Teilnahme!
